# A Self-Compensating Non-Intrusive Ring-Type AC Voltage Sensor Based on Capacitive Coupling

**DOI:** 10.3390/mi15111314

**Published:** 2024-10-29

**Authors:** Junpeng Wang, Jiacheng Li, Chunrong Peng, Zhengwei Wu, Dengfeng Ju, Qiang Zhang

**Affiliations:** 1 State Key Laboratory of Transducer Technology, Aerospace Information Research Institute, Chinese Academy of Sciences, Beijing 100190, China; wangjunpeng22@mails.ucas.ac.cn (J.W.); lijiacheng221@mails.ucas.ac.cn (J.L.); zwwu@mail.ie.ac.cn (Z.W.); 2 School of Electronic, Electrical and Communication Engineering, University of Chinese Academy of Sciences, Beijing 100049, China; 3 Electric Power Intelligent Sensing Technology and Application State Grid Corporation Joint Laboratory, State Grid Smart Grid Research Institute Co., Ltd., Beijing 102209, China; judf321@163.com (D.J.); ncepu_zhangqiang@163.com (Q.Z.)

**Keywords:** non-intrusive measurement, AC voltage, self-compensating, capacitive coupling

## Abstract

In order to reduce the influence of coupling capacitance variations on cable voltage measurement, this paper proposes a self-compensating non-intrusive ring-type AC voltage sensor based on capacitive coupling. A theoretical model of the sensor was established, and the influence of parasitic capacitance changes on sensor output was analyzed. Furthermore, a theoretical analysis shows that the parasitic capacitance between the external cable and the sensing probe, as well as between the ground and the sensing probe, will significantly affect the sensitivity of the sensor and increases the measurement error. A ring-type inductive probe and a signal processing circuit were designed, incorporating a reference signal to compensate for the influence of coupling capacitance variations. Additionally, to minimize the impact of parasitic capacitance on sensor output, the length of the outer ring electrode was extended, and a PTFE housing was designed for protection. A prototype of the sensor was developed and tested. This prototype has a good linear response to AC voltage in the measurement range of 0–1000 V with a linearity of 0.86%. The effects of changes in cable diameter and cable position on the measurement were tested separately. The worst-case error of the sensor output is less than 6.44%, representing a reduction of 21.4% compared to the uncompensated case. Under external cable interference, the sensor exhibited an output error of less than 1.85%. The results show that the designed sensor can accurately measure cable voltage despite changes in cable diameter or installation position, and also demonstrates effective shielding against external interference.

## 1. Introduction

Voltage measurement of the cable plays a pivotal role in the power system, through which the measurement of the cable voltage can realize power grid fault location, condition monitoring, and load assessment [1,2]. The primary sensors utilized for measuring voltage in an electrical grid are voltage transformers and direct-contact meters [3,4,5]. However, both voltage transformers and direct-contact meters require physical contact with the power line, which involves removing the insulating outer layer of the power line [6,7], and this can pose safety risks, particularly in high-voltage situations. Consequently, they may no longer meet the demands of modern power systems.

Non-intrusive voltage measurement allows for the measurement of cable voltage without physical contact, offering advantages in safety, reliability, and operational simplicity. It represents a significant trend in the future development of power system voltage measurement. Non-contact voltage measurements are typically performed by detecting the electric field at a certain distance from the cable to determine the cable voltage. Currently, non-contact voltage measurement mainly includes optical non-intrusive voltage measurements and non-intrusive measurements based on capacitive coupling. Optical non-invasive measurements entail the conversion of the input electrical signal into an optical signal by using the optical anisotropy of an electro-optical crystal [8,9,10]. This optical signal is then converted into an electrical output following transmission over fiber optics. Among these, optical voltage sensors utilizing the Pockels effect are the most prevalent. Optical non-intrusive voltage measurements exhibit high accuracy, but the device is costly, is intricate, and exhibits low reliability [11,12,13]. Therefore, they are not yet suitable for large-scale practical applications.

Capacitive coupling measurements measure cable voltage by detecting the alternating electric field surrounding the cable. Compared to optical measurements, this method offers the advantages of a lower cost and better environmental adaptability [14,15,16,17]. However, variations in cable diameter and position can affect the magnitude of the coupling capacitance between the cable and the sensor, significantly affecting the accuracy of capacitive coupling measurements. To address these shortcomings of capacitive coupling non-intrusive voltage measurements, a study [18] introduces dynamic sampling capacitance to derive the coupling capacitance between the cable and the sensor. While this approach compensates for cable changes, it places high demands on the parameters of the sampling capacitance. A D-dot measurement method is proposed for reconstructing the cable voltage by multiple sensors in the literature [19,20]. This method, however, demands high sensor position accuracy, necessitating the precise determination of cable and sensor coordinates. Additionally, the literature [11,21] achieves self-compensation for cable diameter variations and position variations by introducing an AC reference voltage. Nevertheless, this literature does not account for external cable interference. In summary, current compensation methods have certain limitations, including high equipment requirements, system complexity, and failure to account for external cable interference.

In this paper, a new type of non-intrusive AC cable voltage sensor is proposed. The proposed sensor is based on the principle of capacitive coupling, and a theoretical model is established to support its operation. Compared to existing studies, this paper employs fractional linear transformation to theoretically derive the impact of external cables and other disturbances on measurement errors. The findings indicate that the parasitic capacitance between the external cables and the probe significantly affects measurement accuracy. By improving the probe design and incorporating a polytetrafluoroethylene (PTFE) shell, the influence of parasitic capacitance is effectively mitigated. A prototype of the sensor was developed and experimentally verified, and non-intrusive measurement of cable voltage in the range of 0–1000 V was realized. Test results demonstrate that the sensor can accurately measure cable voltage despite changes in cable diameter or position. Furthermore, the sensor effectively shields against interference from external cables, enabling high-precision measurement of the cable voltage.

## 2. Sensor Design

**Figure 1:**
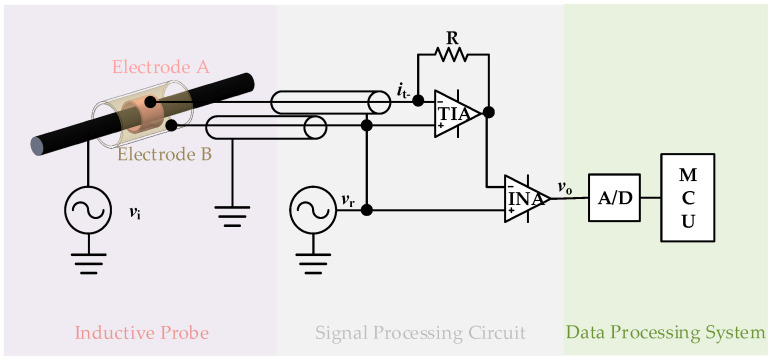
Simplified diagram of self-compensating non-intrusive ring-type AC voltage sensor based on capacitive coupling.

**Figure 2:**
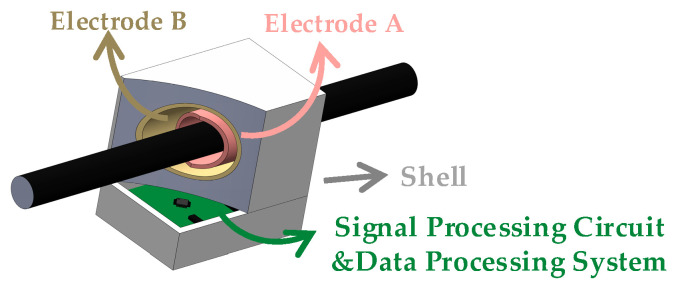
The structure of the sensor.

Figure 1 illustrates a simplified diagram of the self-compensating non-intrusive ring-type AC voltage sensor based on capacitive coupling, designed in this paper. The sensor consists of three principal components: an inductive probe, a signal processing circuit, and a data processing system. The inductive probe features two ring-shaped coaxial electrodes (designated as electrode A and electrode B) and an insulating medium filling the space between the electrodes. According to the theoretical analysis, the length of electrode B is extended to reduce the effect of the principal parasitic capacitance. During the measurement process, the inductive probe can be opened along the radial direction of the hole to allow the cable to be placed into the hole. The signal processing circuit includes a shielded cable, a reference signal generator, a transimpedance amplifier, and an instrumentation amplifier. The reference signal is applied to the noninverting input of the transimpedance amplifier, the noninverting input of the instrumentation amplifier, and electrode B. The data processing system comprises an A/D converter chip and a microcontroller unit (MCU). This system performs the analog-to-digital conversion of the signals, processes them in the MCU using techniques such as a fast Fourier transform (FFT), and finally obtains a cable voltage that is not affected by changes in parasitic capacitance. As illustrated in Figure 2, the system is integrated in a PTFE shell that suppresses interference with measurements due to changes in the external environment.

## 3. Principle

### 3.1. Self-Compensation Principle

**Figure 3:**
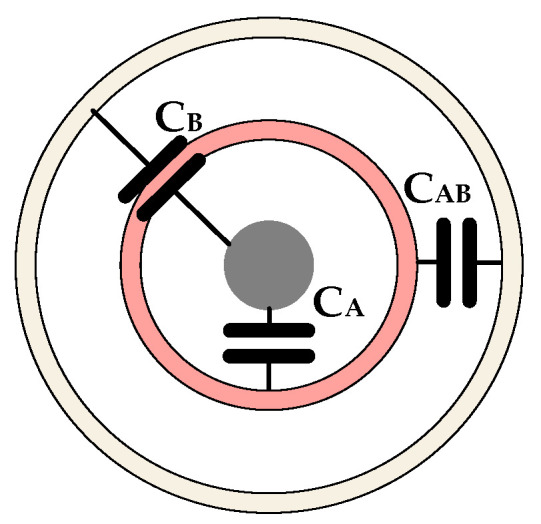
A schematic of capacitance between the cable and the sensing probe.

The sensor described in this paper is based on the principle of capacitive coupling for the non-contact measurement of cable voltage. Without considering parasitic capacitance, two capacitances are present between the cable and the sensing probe: the capacitance $C_{A}$ between the cable and electrode A and the capacitance $C_{B}$ between the cable and electrode B. Additionally, an internal capacitance, $C_{\mathrm{AB}}$, exists between the two electrodes, as illustrated in Figure 3.

Assuming the reference voltage is $v_{r}=V_{r}\sin(\omega_{r}t)$ and the cable voltage is $v_{i}=V_{i}\sin(\omega_{i}t)$ (where $\omega_{i}$ is known), the voltage of electrode A is equal to the reference voltage. So, the current output from the capacitor can be expressed as follows:

(1) $$i_{t-}=C_{A}\frac{d}{dx}(V_{r}\sin(\omega_{r}t)-V_{i}\sin(\omega_{i}t))=j\omega_{r}C_{A}[V_{r}\cos(\omega_{r}t)-V_{i}\cos(\omega_{i}t)]$$

Therefore, the output of the instrumentation amplifier can be given by

(2) $$v_{o}=G[j\omega_{r}RC_{A}V_{r}\cos(\omega_{r}t)-j\omega_{i}RC_{A}V_{i}\cos(\omega_{i}t)]$$

where G is the instrumentation amplifier gain and R is the transimpedance amplifier gain resistor. Subsequently, the $v_{o}$ is transferred to the MCU following the ad conversion process. An FFT is then performed to extract the cochannel signals at the $\omega_{r}$ and $\omega_{i}$ frequencies: $v_{\mathrm{or}}=G\omega_{r}RCV_{r},v_{\mathrm{oi}}=G\omega_{i}RCV_{i}$.

Therefore, the voltage of the cable is given by

(3) $$V_{i}=\frac{V_{\mathrm{oi}}}{V_{\mathrm{or}}}\frac{\omega_{r}}{\omega_{i}}V_{r}$$

It can be observed that the output processed by the MCU is not influenced by the capacitance $C_{A}$. This ensures that variations in cable diameter and position will not affect the accuracy of the voltage measurement.

### 3.2. Effect of Parasitic Capacitance

**Figure 4:**
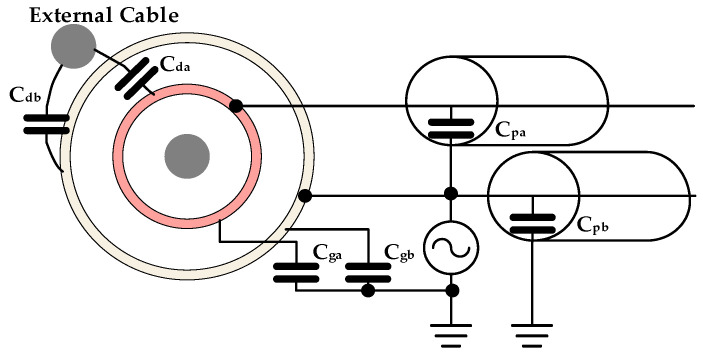
Schematic of parasitic capacitance.

The above analysis is based on ideal conditions. In actual measurements, the parasitic capacitances between the external cable and the probe, between the ground and the probe, and within the measurement system can all influence the measurement output. The measurement system considering parasitic capacitance is shown in Figure 4. $C_{\mathrm{da}}$ and $C_{\mathrm{db}}$ are the capacitances between the external cable and the electrodes; $C_{\mathrm{ga}}$ and $C_{\mathrm{gb}}$ are the capacitances between the ground and the electrodes; and $C_{\mathrm{pa}}$ and $C_{\mathrm{pb}}$ are the capacitances between the center of the shielded cable and its outer layer.

The effect of $C_{\mathrm{pa}}$ is negligible as the potentials at electrodes A and B are the same. $C_{\mathrm{db}}$, $C_{\mathrm{gb}}$, and $C_{\mathrm{pb}}$ will affect the reference voltage. However, this effect is negligible due to the low output impedance of $v_{r}$. On the other hand, $C_{\mathrm{da}}$ and $C_{\mathrm{ga}}$ are equivalent to a parallel connection with $C_{A}$, which will have a significant effect on the measurement output. It is therefore necessary to investigate the effect of $C_{\mathrm{da}}$ and $C_{\mathrm{ga}}$ on the output.

Suppose the external cable signal is $v_{d}=V_{d}\sin(\omega_{d}t)$. The output of the instrumentation amplifier is corrected by the following equation:

(4) $$\begin{array}{ccc} v_{o} & = & G[j\omega_{r}RCV_{r}\cos(\omega_{r}t)-j\omega_{i}RCV_{i}\cos(\omega_{i}t)+j\omega_{r}RC_{\mathrm{ga}}V_{r}\cos(\omega_{r}t)\\ & & +j\omega_{r}RC_{\mathrm{da}}V_{r}\cos(\omega_{r}t)-j\omega_{d}RC_{\mathrm{da}}V_{d}\cos(\omega_{d}t)] \end{array}$$

Considering that the external cable signal frequency is the same as the cable signal frequency to be measured, the output of the MCU processing is given by

(5) $$V_{i}=\frac{V_{\mathrm{oi}}}{V_{\mathrm{or}}}\frac{\omega_{r}}{\omega_{i}}V_{r}+\frac{V_{oi}}{V_{or}}\frac{\omega_{r}}{\omega_{i}}\frac{C_{\mathrm{ga}}+C_{\mathrm{da}}}{C_{A}}V_{r}-\frac{C_{\mathrm{da}}}{C_{A}}V_{d}$$

It can be seen that the ratio of $\left(C_{\mathrm{ga}}+C_{\mathrm{da}}\right)$ and $C_{A}$ determines the magnitude of the measurement error. Therefore, the magnitudes of $C_{A}$, $C_{\mathrm{ga}}$, and $C_{\mathrm{da}}$ are explored next.

**Figure 5:**
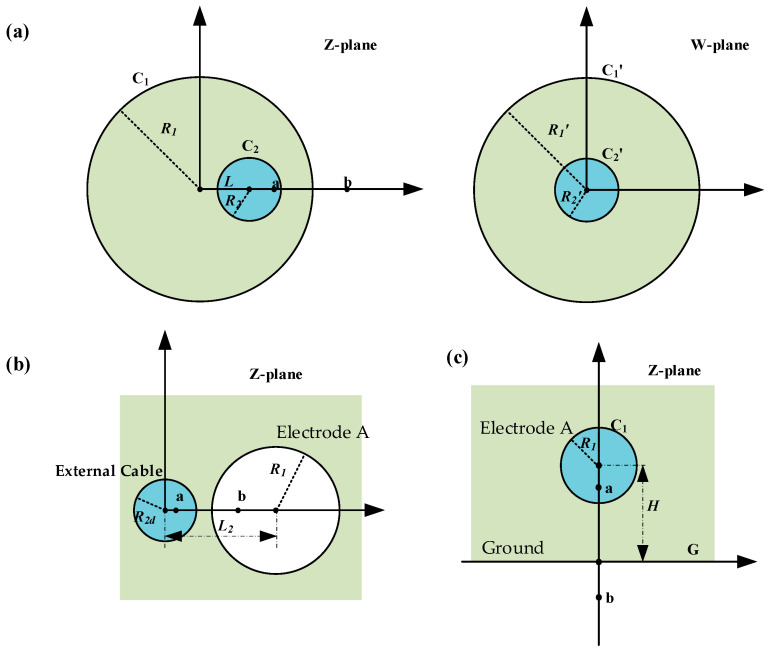
(**a**) The cross-section of the cable and electrode A in the Z-plane the cross-section of the cable and electrode A in the W-plane; (**b**) the cross-section of the external cable and electrode A in the Z-plane; (**c**) the cross-section of the ground and electrode A in the Z-plane.

As shown in Figure 5a, the cross-section of the cable and electrode A is taken. Where circle $C_{1}$ is electrode A, circle $C_{2}$ is the cable and the distance between the centers of the two circles is L. Neglecting the edge effect at the end of electrode A, the solution for $C_{A}$ can be transformed into the solution for the capacitance value between $C_{1}$ and $C_{2}$. The capacitance between two circles with different centers is difficult to solve directly. Therefore, a fractional linear transformation can be performed to transform it from two non-concentric circles in the Z-plane to two concentric circles in the W-plane and then solved [22,23]. The specific steps are as follows.

The center of circle $C_{1}$ is taken as the origin of the Z-plane, and the line joining the centers of the two circles is taken as the *x*-axis. To transform $C_{1}$ and $C_{2}$ into concentric circles, it is first necessary to identify the common symmetry points a ($x_{1}$, 0) and b ($x_{2}$, 0) of the two circles. By the definition of a symmetry point, the following can be obtained:

(6) $$\left\{\begin{array}{c} x_{1}x_{2}=R_{1}^{2}\\ (L-x_{1})(L-x_{2})=R_{2}^{2} \end{array}\right.$$

where $R_{1}$ is the radius of electrode A and $R_{2}$ is the radius of the cable. It can be solved as follows:

(7) $$\left\{\begin{array}{c} x_{1}=\frac{1}{2L}[(L^{2}+R_{1}^{2}-R_{2}^{2})-\sqrt{{\left(L^{2}-R_{1}^{2}-R_{2}^{2}\right)}^{2}-4R_{1}^{2}R_{2}^{2}}]\\ x_{2}=\frac{1}{2L}[(L^{2}+R_{1}^{2}-R_{2}^{2})+\sqrt{{\left(L^{2}-R_{1}^{2}-R_{2}^{2}\right)}^{2}-4R_{1}^{2}R_{2}^{2}}] \end{array}\right.$$

We performed a fractional linear transformation as shown in Equation (8). This transformation maps point a into the origin of the W-plane, while point b is mapped to the point at infinity (∞, 0) in the W-plane. The circle $C_{1}$ is transformed into the circle designated as $C_{1^{\prime }}$ in the W-plane. The circle $C_{2}$ is transformed into the equivalent circle, $C_{2^{\prime }}$, in the W-plane.

(8) $$w\left(z\right)=\frac{z-x_{1}}{z-x_{2}}$$

To calculate the capacitance $C_{A}$, it is also necessary to know the radii $R_{1}^{\prime }$ and $R_{2}^{\prime }$ of the circles $C_{1^{\prime }}$ and $C_{2^{\prime }}$. We took a point $z=R_{1}$ on $C_{1}$, and transformed it to the W-plane with the following equation:

(9) $$w(-R_{1})=\frac{-R_{1}-x_{1}}{-R_{1}-x_{2}}=\frac{R_{1}+x_{1}}{R_{1}+x_{2}}$$

Thus, $R_{1}^{\prime }$ can be obtained as follows:

(10) $$\begin{array}{cc} R_{1}^{\prime } & =\left|\frac{R_{1}+x_{1}}{R_{1}+x_{2}}\right|\\ & =\frac{{\left(\begin{array}{c} L+R_{1} \end{array}\right)}^{2}-R_{2}^{2}-\sqrt{{\left(\begin{array}{c} L^{2}-R_{1}^{2}-R_{2}^{2} \end{array}\right)}^{2}-4R_{1}^{2}R_{2}^{2}}}{{\left(\begin{array}{c} L+R_{1} \end{array}\right)}^{2}-R_{2}^{2}+\sqrt{{\left(\begin{array}{c} L^{2}-R_{1}^{2}-R_{2}^{2} \end{array}\right)}^{2}-4R_{1}^{2}R_{2}^{2}}} \end{array}$$

Similarly, taking a point $z=L+R_{2}$ on the circle $C_{2^{\prime }}$, there are

(11) $$\begin{array}{cc} R_{2}^{\prime } & =\left|\frac{L+R_{2}-x_{1}}{L+R_{2}-x_{2}}\right|\\ & =\frac{{\left(\begin{array}{c} L+R_{2} \end{array}\right)}^{2}-R_{1}^{2}+\sqrt{{\left(\begin{array}{c} L^{2}-R_{1}^{2}-R_{2}^{2} \end{array}\right)}^{2}-4R_{1}^{2}R_{2}^{2}}}{{\left(\begin{array}{c} L+R_{2} \end{array}\right)}^{2}-R_{1}^{2}-\sqrt{{\left(\begin{array}{c} L^{2}-R_{1}^{2}-R_{2}^{2} \end{array}\right)}^{2}-4R_{1}^{2}R_{2}^{2}}} \end{array}$$

For a circular capacitor with the same center, the capacitance is given by the following [24]:

(12) $$C=\frac{\varepsilon_{0}2\pi }{\ln(R_{1}^{\prime }/R_{2}^{\prime })}$$

Consequently, the capacitance between the cable and electrode A can be calculated as follows:

(13) $$C_{A}=\frac{\varepsilon_{0}2\pi }{\ln\left[\frac{R_{1}^{2}+R_{2}^{2}-L^{2}}{2R_{1}R_{2}}+\sqrt{{\left(\frac{R_{1}^{2}+R_{2}^{2}-L^{2}}{2R_{1}R_{2}}\right)}^{2}-1}\right]}$$

Referring to the above process, Figure 5b,c are solved separately. $C_{da}$ and $C_{ga}$ are given by

(14) $$\left\{\begin{array}{l} C_{da}=\frac{\varepsilon_{0}2\pi }{\ln\left[\frac{L^{2}-R_{1}^{2}-R_{2d}^{2}}{2R_{1}R_{2d}}+\sqrt{{\left(\frac{L^{2}-R_{1}^{2}-R_{2d}^{2}}{2R_{1}R_{2d}}\right)}^{2}-1}\right]}\\ C_{ga}=\frac{2\pi \varepsilon_{0}}{\ln\left(\frac{H}{R_{1}}+\sqrt{\frac{H^{2}}{R_{1}^{2}}-1}\right)} \end{array}\right.$$

Substituting the parameters from Table 1 into the calculation, there is

(15) $$\frac{C_{\mathrm{ga}}}{C_{A0}}=0.3038,\frac{C_{\mathrm{da}}}{C_{A0}}=0.3363$$

where $C_{A0}$ is the capacitance between the cable and electrode A calculated from Table 1. It is evident that in this instance, there is a discrepancy of at least 61% between the output value and the actual value. This will have a significant impact on the self-compensation effect in practical measurements. The effect of changes in wire and cable diameters and positions on $C_{A}$ is calculated.

**Figure 6:**
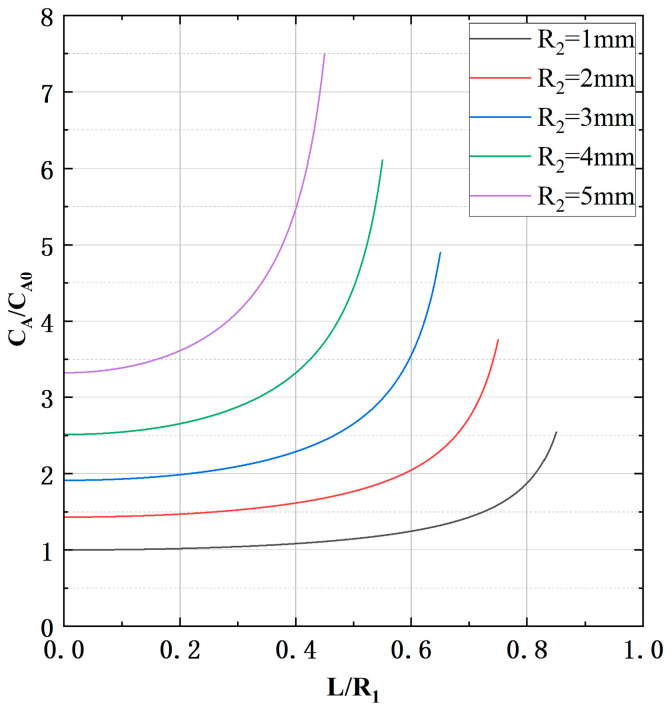
The relationship between $C_{A}$ and L for different $R_{2}$.

As shown in Figure 6, $C_{A}$ increases with increasing cable diameter ($R_{2}$). In addition, as the cable position offset (L) increases, the value of $C_{A}$ increases exponentially, further increasing the measurement error. In this paper, by increasing the length of electrode B and correspondingly reducing the length of electrode A, most of the capacitance from electrode A to the external cable and ground is converted to capacitance from electrode A to electrode B. This reduces $C_{\mathrm{ga}}$ and $C_{\mathrm{da}}$, and thus the measurement error.

## 4. Experimental Setup and Results

### 4.1. Experimental Setup

**Figure 7:**
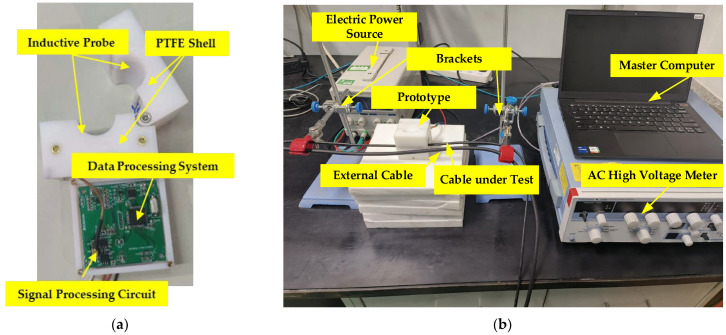
(**a**) Photograph of prototype; (**b**) photo of testing system.

Figure 7a shows a photograph of the prototype self-compensating non-intrusive ring-type AC voltage sensor designed in this paper. The prototype is housed in a PTFE shell. Electrodes A and electrodes B of the probe are made of copper and connected to the signal processing circuit by shielded cable. To improve measurement accuracy, the frequency of the reference signal should be significantly higher than that of the cable under testing. However, due to the limitation of the circuit bandwidth, the reference signal frequency is set to 3000 Hz. The schematics of the signal processing circuit and the data processing system are shown in Figure 1. The TIA of the signal processing circuit is realized using a low-offset AD8626 from Analog Devices, Wilmington, MA, USA, and the INA of the signal processing circuit is implemented using a high-accuracy AD620 also from Analog Devices, Wilmington, MA, USA. The ADC of the data processing system is realized using an AD7606 from Analog Devices, Wilmington, MA, USA, and the MCU of the data processing system is realized using an STM32F405 from STMicroelectronics, Plan-les-Ouates, Geneva, Switzerland. Figure 7b shows a photo of the testing system, which consists of six parts: the cable under testing, the external cable, the electric power source, the brackets, the AC high-voltage meter, and the master computer. The brackets allow adjustment of the cable’s position within the probe. The AC high-voltage meter is employed for the generation of voltages with varying frequencies and amplitudes. The device is capable of generating AC voltages between 0 and 1000 Vrms with an accuracy of 0.04% and a frequency range of 40 to 1000 Hz. The master computer is employed to receive the signals that have been processed by the MCU.

### 4.2. Linearity Test

**Figure 8:**
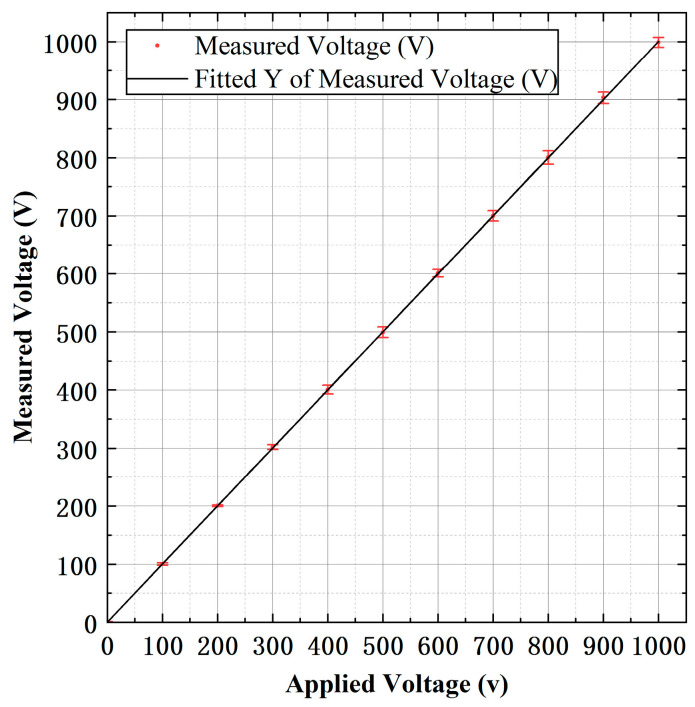
Prototype response to AC applied voltages between 0 and 1000 V at 50 Hz.

In order to verify the accuracy of the voltage measurement, the prototype was tested for linearity. Tests were conducted at a range of AC voltages between 0 and 1000 V at 50 Hz. Figure 8 shows the response of the prototype to AC applied voltages between 0 and 1000 V at 50 Hz with error bars. The best fit straight line between the output voltages from MCU and the applied voltages is $V_{mea}=0.999\times V_{app}+0.927$, and a linearity of 0.86% is obtained.

### 4.3. Response to AC Voltage

**Figure 9:**
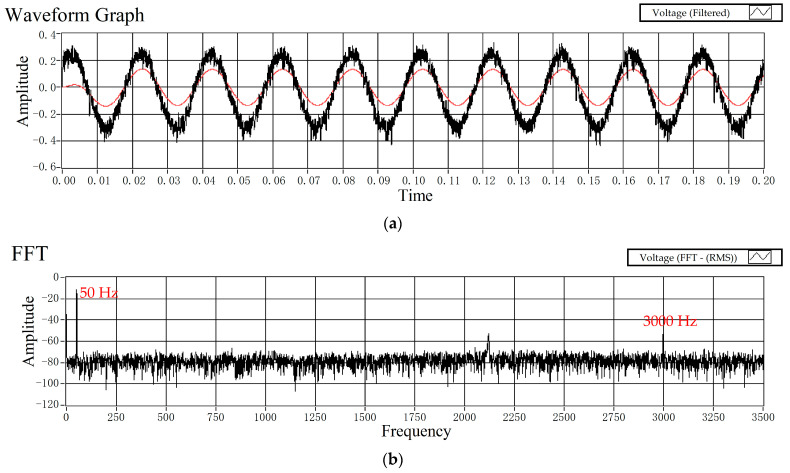
(**a**) The prototype response to the AC input voltage (50 Hz); (**b**) the spectrum of the analog signal output by the signal processing circuit.

To evaluate the capacity of the prototype to measure AC waveform signals, measurement of the instantaneous input signal at the power frequency was conducted. The input signal was acquired using a resistive voltage divider for waveform comparison. The output of the signal processing circuit is illustrated in Figure 9a. It can be observed that the sensor exhibits a good response to the transient waveform of the AC input signal. Figure 9b illustrates the spectrum of the analog signal output by the signal processing circuit. It can be seen that there are two frequency components in the voltage signal of the sensor output, which are 50 Hz and 3000 Hz ($\omega_{i}$).

### 4.4. Self-Compensation

The self-compensation of the prototype was then tested. With the 50 Hz AC voltage constant at 233 V, the capacitance $C_{A}$ was varied by varying the cable diameter and changing the cable position to test the compensation effect of the prototype output. Keeping the cable centered, tests were conducted using wires of different diameters. The results of this test are presented in Table 2. Comparing the output with the reference signal applied and the output with the reference signal grounded, it is observed that the introduction of a reference signal for self-compensation reduced the measurement error by a maximum of 21.4%.

Next, keeping the diameter of the cable constant (9 mm), the position of the cable was changed for testing; the results are shown in Table 3. The maximum error of the prototype output when the cable position was changed is 5.15%. Consequently, the prototype designed in this paper is capable of effectively compensating for alterations in cable diameter and position, thereby ensuring the precise measurement of cable voltage.

### 4.5. Shielding Effect

**Figure 10:**
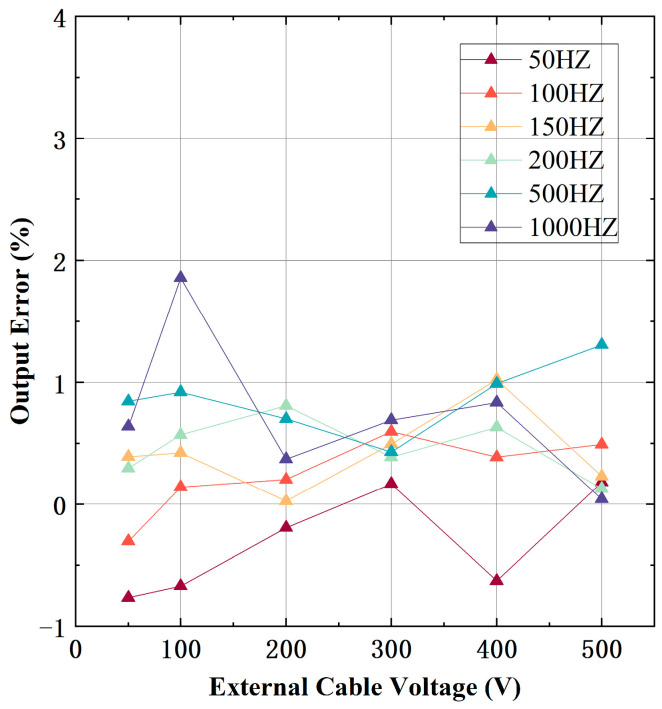
The output error of the prototype under external cable interference with the external cable voltage ranging from 50 to 500 V and the frequency ranging from 50 to 1 kHz.

To verify the shielding effect of the prototype, an external cable was placed beside the side of the inductive probe. A constant voltage of 220 V at 50 Hz was applied to the cable under testing. To evaluate the prototype’s output under different magnitudes and frequencies of external interference, an interference signal with a range of 50 to 500 V and 50 to 1000 Hz was applied to the external cable. The output error of the prototype under external cable interference, as shown in Figure 10, exhibits a maximum error of only 1.85%. It can be seen that by extending the length of electrode B and designing the PTFE shell, the prototype designed in this paper can accurately measure the voltage of the cable under testing, even in the presence of external cable interference.

## 5. Conclusions

In this paper, a theoretical model is developed to derive the coupling capacitance variations and the parasitic capacitance affecting the sensor output. The effect of changes in wire diameter and cable position on the measurement is analyzed by theoretical calculations. Additionally, the effect of parasitic capacitance between the external cable and the sensing probe, as well as between the ground and the sensing probe, on the sensor output has been investigated. By designing a ring-type sensing probe and introducing a reference signal, the effect of changes in wire diameter and cable position on the measurement was reduced. Furthermore, the length of the outer ring electrode was extended and a PTFE shell was designed in order to reduce the effect of the main parasitic capacitance on the sensor output. The prepared prototype achieved 0–1000 V AC voltage measurement with good linearity (0.86%). The effects of changes in cable diameter and cable position on the measurement were tested separately. The worst-case error of the sensor output is less than 6.44%, representing a reduction of 21.4% compared to the uncompensated case. Under external cable interference, the sensor exhibited an output error of less than 1.85%. The anti-interference characteristics of the sensor will be further investigated in the future to optimize measurement error of the sensor for existing measurement requirements.

## Figures and Tables

**Table 1:** Typical parameters of the structure.

Symbols	Value	Symbols	Value
$R_{1}$	10 mm	$L_{2}$	50 mm
$R_{2}$	2 mm	$R_{2d}$	2 mm
L	0 mm	H	1000 mm

**Table 2:** Different cable diameter test results.

Cable Diameter (mm)	Measured Voltage Without Self-Compensation (V)	Relative Deviation (%)	Measured Voltage with Self-Compensation (V)	Relative Deviation (%)
1.5	267	14.60	248	6.44
2	196	15.64	246	5.58
3	284	21.83	234	0.43
4	265	13.87	235	0.86
9	294	26.21	221	5.15

**Table 3:** Different cable position offset test results.

Cable Position Offset (mm)	Measured Voltage with Self-Compensation (V)	Relative Deviation (%)
0	221	5.15
2	228	1.94
4	237	1.83
6	222	4.50

## Data Availability

Data are contained within the article.

## References

[1] Yang P., Wen X., Lv Y., Chu Z., Peng C. (2021). A Non-Intrusive Voltage Measurement Scheme Based on MEMS Electric Field Sensors: Theoretical Analysis and Experimental Verification of AC Power Lines. Rev. Sci. Instrum..

[2] Liu J., Xia S., Peng C., Chu Z., Lei H., Liu X., Zhang Z., Zhang W., Peng S., Gao Y. (2023). A Wafer-Level Vacuum Packaged MEMS Electric Field Sensor Based on SOI-SOG Bonding. Acta Electron. Sin..

[3] Mohanty R., Pradhan A.K., Dutta P.K. (2024). Accurate Voltage Phasor Measurement for Electric Power Transmission Line Protection Using Current Sensor. IEEE Sens. Lett..

[4] Krause T.C., Camenzind K., Green D.H., Moeller A., Huchel L., Leeb S.B. (2022). A Sensor Topology for Noncontact AC Voltage Measurement of Polyphase Cables. IEEE Trans. Instrum. Meas..

[5] Sun S., Ma F., Yang Q., Ni H., Bai T., Ke K., Qiu Z. (2023). Research on Non-Contact Voltage Measurement Method Based on Near-End Electric Field Inversion. Energies.

[6] Xing Y., Liu J., Li F., Zhang G., Li J. (2023). Advanced Dual-Probes Noncontact Voltage Measurement Approach for AC/DC Power Transmission Wire Based on the Electric Field Radiation Principle. IEEE Trans. Instrum. Meas..

[7] Haberman M.A., Spinelli E.M. (2020). A Noncontact Voltage Measurement System for Power-Line Voltage Waveforms. IEEE Trans. Instrum. Meas..

[8] Wang H., Zeng R., Zhuang C., Lyu G., Yu J., Niu B., Li C. (2020). Measuring AC/DC Hybrid Electric Field Using an Integrated Optical Electric Field Sensor. Electr. Power Syst. Res..

[9] Wu Q., Zhang X.C. (1996). Ultrafast Electrooptic Field Sensors. Appl. Phys. Lett..

[10] Zeng R. (2014). Study of an Integrated Optical Sensor with Mono-Shielding Electrode for Intense Transient E-Field Measurement. Measurement.

[11] Shenil P.S., George B. An Auto-Balancing Scheme for Non-Contact AC Voltage Measurement. Proceedings of the 2018 IEEE 9th International Workshop on Applied Measurements for Power Systems (AMPS).

[12] Shenil P.S., Arjun R., George B. Feasibility Study of a Non-Contact AC Voltage Measurement System. Proceedings of the 2015 IEEE International Instrumentation and Measurement Technology Conference (I2MTC) Proceedings.

[13] Haberman M.A., Spinelli E.M. (2018). Noncontact AC Voltage Measurements: Error and Noise Analysis. IEEE Trans. Instrum. Meas..

[14] Martins A.V., Bacurau R.M., Dos Santos A.D., Ferreira E.C. (2020). Nonintrusive Energy Meter for Nontechnical Losses Identification. IEEE Trans. Instrum. Meas..

[15] Gorla D.P.M., Janus P., Edin H. Non-Contact Voltage Measurement Technique for On-Line Monitoring of Transient Overvoltages. Proceedings of the Nordic Insulation Symposium.

[16] Pouryazdan A., Costa J.C., Prance R.J., Prance H., Munzenrieder N. Non-Contact Long Range AC Voltage Measurement. Proceedings of the 2019 IEEE SENSORS.

[17] Suo C., Huang R., Zhou G., Zhang W., Wang Y., He M. (2023). Self-Calibration Sensor for Contactless Voltage Measurement Based on Dynamic Capacitance. Sensors.

[18] Suo C., He M., Zhou G., Shi X., Tan X., Zhang W. (2023). Research on Non-Invasive Floating Ground Voltage Measurement and Calibration Method. Electronics.

[19] Si D., Wang J., Wei G., Yan X. (2020). Method and Experimental Study of Voltage Measurement Based on Electric Field Integral With Gauss–Legendre Algorithm. IEEE Trans. Instrum. Meas..

[20] Jakubowski J., Kuchta M., Kubacki R. (2021). D-Dot Sensor Response Improvement in the Evaluation of High-Power Microwave Pulses. Electronics.

[21] Shenil P.S., George B. (2019). Development of a Nonintrusive True-RMS AC Voltage Measurement Probe. IEEE Trans. Instrum. Meas..

[22] Palmer H.B. (1937). The Capacitance of a Parallel-Plate Capacitor by the Schwartz-Christoffel Transformation. Trans. Am. Inst. Electr. Eng..

[23] Cohn S.B. (1955). Problems in Strip Transmission Lines. IEEE Trans. Microw. Theory Techn..

[24] Yang P., Wen X., Chu Z., Ni X., Peng C. (2021). Non-Intrusive DC Voltage Measurement Based on Resonant Electric Field Microsensors. J. Micromech. Microeng..

